# Scalable, full-colour and controllable chromotropic plasmonic printing

**DOI:** 10.1038/ncomms9906

**Published:** 2015-11-16

**Authors:** Jiancai Xue, Zhang-Kai Zhou, Zhiqiang Wei, Rongbin Su, Juan Lai, Juntao Li, Chao Li, Tengwei Zhang, Xue-Hua Wang

**Affiliations:** 1 State Key Laboratory of Optoelectronic Materials and Technologies, Sun Yat-sen University, Guangzhou 510275, China; 2 School of Physics and Engineering, Sun Yat-sen University, Guangzhou 510275, China

## Abstract

Plasmonic colour printing has drawn wide attention as a promising candidate for the next-generation colour-printing technology. However, an efficient approach to realize full colour and scalable fabrication is still lacking, which prevents plasmonic colour printing from practical applications. Here we present a scalable and full-colour plasmonic printing approach by combining conjugate twin-phase modulation with a plasmonic broadband absorber. More importantly, our approach also demonstrates controllable chromotropic capability, that is, the ability of reversible colour transformations. This chromotropic capability affords enormous potentials in building functionalized prints for anticounterfeiting, special label, and high-density data encryption storage. With such excellent performances in functional colour applications, this colour-printing approach could pave the way for plasmonic colour printing in real-world commercial utilization.

## Abstract (web summary)

Plasmonics offers an alternative approach to robust colour printing, but real applications require a full-colour and scalable process. Here, the authors develop such an approach by combining plasmonic broadband absorbers with conjugate twin-phase modulation, demonstrating its potential for anticounterfeiting technology.

## Graphical abstract

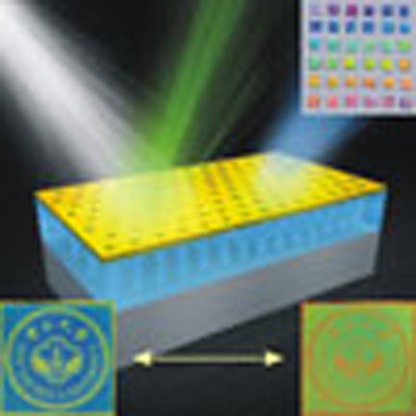

Surface plasmons (SP), collective oscillations of free electrons excited by light, possess the intrinsic ability of resonant interaction with electromagnetic waves, which leads to their unique capability for preferential photon absorption, selective scattering or diffraction^1^,^2^,^3^. These properties have inspired the field of plasmonic colour applications, which has advantages such as high compactness, robust stability, high tunability and compatibility of functionalization^4^,^5^,^6^,^7^,^8^,^9^,^10^,^11^,^12^,^13^,^14^. Numerous promising advanced devices have been cultivated including simple filters as well as multifunctional devices, such as full colour filters and polarizers^4^,^5^,^6^, macroscopic colour holograms^7^, CMOS image sensors^8^ and functional plasmonic gratings^9^ for transmitted plasmonic colour devices, versatile reflective filters^10^, electrochromic devices^11^ and colour printing and display^12^,^13^,^14^ in reflective type. These achievements in plasmonic colour applications not only expand our understanding of nano-optics, but also enrich our life with vivid colour, giving rise to considerate benefits in both fundamental science and applied technologies.

In the exploration of various plasmonic colour applications, a widely studied and distinctive research emphasis is on plasmonic colour printing because of its great potential for the next-generation colour-printing technology. Due to the impressive advantages it has demonstrated in high-resolution display^13^,^14^, consumer product colouration^15^, image reproducing^16^ and functionalized colour printing^17^, plasmonic colour printing exhibits superiority in developing high-density optical data storage, colour image display, commercial anticounterfeiting and data encryption. Beyond the fundamental studies, now research in plasmonic colour printing has been focused on the experimental attempts to convert its potentials into real applications, where two major issues must be addressed: full colour generation and scalable fabrication.

In terms of full colour generation, displaying vivid colours ranging from blue to red requires distinct reflective peak in reflection spectra. However, the displayed colours of most plasmonic printing techniques are the complementary colours of the reflective valley^13^,^14^,^15^,^17^,^18^,^19^, which limits their ability to achieve full colour printing. To date, only two works have been able to generate plasmonic reflective colours through the formation of reflective peaks^12^,^20^, indicating the possibility of full colour printing. Furthermore, most plasmonic printing approaches, including those that demonstrate the potential for full-colour printing, involve electron-beam lithography (EBL), a technique that is not only complex and expensive, but also limited in the sample size. Therefore, it is highly desired to develop an efficient approach that can realize full colour and scalable fabrication in the field of plasmonic colour printing.

On the other hand, demands for functional colour devices are rapidly growing with applications such as anticounterfeiting devices, security tags and functionalized decoration being investigated^6^,^21^,^22^,^23^. So far plasmonic colour-printing technology has yet to contribute to this newly arising field of functionalized colour application. But this technology is believed to possess great potential in constructing such functional devices because of its unique and unexplored advantages. For example, the controllable chromotropic capability^24^,^25^, that is, the ability of reversible colour transformations with on-demand colour output, can be a key breakthrough point that deserves research efforts and application attempts.

In the following, we describe an approach that overcomes the aforementioned challenges, using conjugate twin-phase modulation (CTPM) to maximize the peak–valley (p–v) ratio of reflection spectra, and introducing metal island film as a plasmonic broadband absorber (PBA) to narrow the reflective peaks. This combination successfully generates vivid full colour. In addition, a sputtered metal island film and electrochemical grown anodic alumina oxide (AAO) template are utilized to construct plasmonic prints that scale up to centimetre sizes. Taking advantage of the AAO template, we demonstrate the chromotropic capacity in the field of plasmonic colour printing, and exhibit its potentials for functional applications in commercial pattern colouration, anticounterfeiting labels and high-density data encryption storage. Furthermore, instead of gold or silver, we prove that all these functionalities can be implemented with aluminum, the third most abundant element on earth, which is low-cost, durable and suitable for industrial applications. Our work not only presents theoretical and experimental methods for full-colour plasmonic printing, but also provides a practical avenue to the applications of plasmonic colour devices in commercial utilizations.

## Results

### Mechanism of CTPM–PBA colour generation

**Figure 1:**
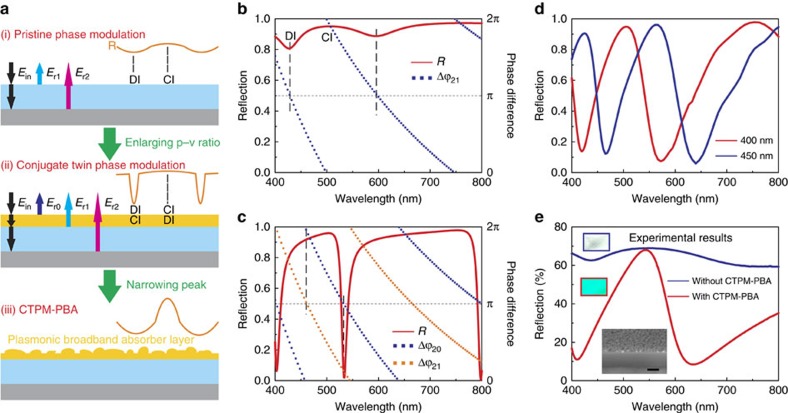
Mechanism. (**a**) Illustration of the CTPM–PBA mechanism. (i) The pristine phase modulation in a thin dielectric film on an opaque substrate. DI and CI between *E*_r1_ and *E*_r2_ render vague reflective valleys and peaks, respectively. (ii) Enlarging p–v ratio by the asymmetric phase modulation with a thin metal coating. Wavelengths of CI and DI in the metal layer conversely correspond to that of DI and CI of the reflected waves at the outer surface, respectively, resulting in distinct reflective valleys as well as wide and flat peaks. (iii) Narrowing reflective peaks by CTPM–PBA with a metallic island film. The metallic island film works as a PBA, making the reflective peaks much narrower. (**b**) Calculated spectra in the pristine phase modulation of a 400-nm-thick Al_2_O_3_ (*n*=1.76) on silver substrate. Small reflective valleys and peaks appear when Δ*ϕ*_21_ (the phase difference between *E*_r1_ and *E*_r2_) equals to 0 (or 2*π*) and *π*, respectively. (**c**) Calculated spectra in the asymmetric phase modulation of the structure in **b** with a 30-nm-thick plain coating of silver. Distinct valleys occur when Δ*ϕ*_20_≈π (DI), while Δ*ϕ*_21_≈0 or 2π (CI in the metal layer). (**d**) Simulated reflective spectra of Al_2_O_3_ thin-film coated with a silver island film (average thickness equals to 8 nm) on silver substrate. Narrow reflective peaks appear in spectra corresponding to Al_2_O_3_ films with thickness of 400 nm (red curve) and 450 nm (blue curve). (**e**) Experimental demonstration of the CTPM–PBA colour generation. The 360-nm-thick SiO_2_ film on aluminum substrate had very low p–v ratio in reflective spectra (blue curve) and no distinct colour (insert with blue square), but narrow peak with large p–v ratio (red curve) and vivid cyan colour (insert with red square) appeared after coated with a silver island film. The bottom insert is the SEM morphology of the sample with vivid cyan, observed in a tilted angle of 45°. Scale bar, 200 nm. The theoretical spectra were calculated in normal incidence, and the experimental results were measured with the incident angle of 8°.

Generally, for a transparent dielectric film on an opaque substrate, the optical phase modulation of the light wave in the structure (shown in Fig. 1a(i)) can shape the reflection spectra into a specific profile with fluctuations. This simple case can be easily understood in the way of thin-film interference, where the reflective valleys form because the reflected light waves from the outer (*E*_1r_) and inner (*E*_2r_) interface are out of phase, that is, the phase difference between *E*_1r_ and *E*_2r_ (Δ*ϕ*_21_) equals to *π* (destructive interference, DI); while reflective peaks appear at wavelengths corresponding to in-phase light waves (Δ*ϕ*_21_=0 or 2*π*) and constructive interference (CI; Fig. 1a(i), Supplementary Fig. 1 and Supplementary Note 1). As this spectral fluctuations fall into the visible spectrum, they create the possibility for colour display. Nevertheless, distinct colour output requires maximized reflective p–v ratio (that is, the ratio of the peak reflection to the valley reflection) to show high saturation, which cannot be achieved by the pristine phase modulation with this one-layer film (Fig. 1b).

If a thin metal layer is coated on the top of the transparent dielectric film and the largest light absorption can be achieve within the metal layer at the wavelengths of the reflective valleys, then the reflective p–v ratio can be maximized. This requires realizing the CTPM, which means the simultaneous occurrence of both the DI of the reflected waves on the outer-interface of the thin metal layer and the CI in the thin metal layer at the same wavelength. This implies that the phase difference Δ*ϕ*_21_ between *E*_1r_ and *E*_2r_ equals to 0 or 2*π* (rather than the aforementioned *π* phase difference) and the phase difference Δ*ϕ*_01_ (Δ*ϕ*_02_) between *E*_0r_ and *E*_1r_ (*E*_2r_) must be *π*. Figure 1a(ii), Supplementary Fig. 2, Supplementary Fig. 3 and Supplementary Note 2 clearly explains the origins of the CTPM and how it is realized.

In this work, we will apply the physical mechanism of the CTPM to optimize the structure, in which the thickness of the dielectric is determined by Δ*ϕ*_21_=0 or 2*π*, and that of the metal layer is designed to make both the propagating phase of light negligible and its absorption sufficient in the metal layer. The calculated reflection spectrum in Fig. 1c proves the validity of the CTPM in maximizing the p–v ratio. It is worth pointing out that the CTPM requires the propagating phase of light in the metal layer to be negligible, therefore, the thickness of the metal layer should be as thin as possible. However, if the layer is too thin, sufficient light absorption cannot be achieved and the p–v ratio will not be significantly enhanced. The relationship between the reflection spectra and the Ag layer thickness is discussed in Supplementary Fig. 4, which enables the determination of an approximate thickness.

In addition to the maximized p-v ratio, full-colour plasmonic printing also requires sharp reflective peaks. However, the CTPM can only bring about very narrow-band absorption in the plain metal film nearby the reflective valleys, forming very wide and flat reflective peaks (Supplementary Figs 3a and 5a). In order to narrow the reflective peaks, it is necessary to produce strong light absorption within a wide spectral range around the reflective valleys. To address this issue, we replaced the plain metal film with a metallic island film working as PBA^26^,^27^, which exhibited high absorption efficiency through the entire visible spectrum. In our experiments, the PBA successfully narrowed and sharpened the reflective peaks significantly (Fig. 1d, Supplementary Figs 5 and 6). Without such a PBA layer, both the saturation and the hue were seriously limited^28^,^29^. The broadband absorption property of the metallic island film is attributed to its unique morphology, containing metal nano-particles with different sizes and shapes that lead to different plasmonic resonant wavelengths and diffusion scattering, and consequently form the apparent plasmonic broadband absorption. Supplementary Figs 5b, 7 and 8 give a more detailed discussion. Moreover, this effect is universal for different kinds of dielectric layers and substrates (Supplementary Fig. 9). As an experimental demonstration, we deposited a thin SiO_2_ film on a polished aluminum substrate, and a rough silver layer was then sputtered onto the sample to show vivid colour (Fig. 1e). The discussion about the order of the multiple peaks in reflective spectra can be found in Supplementary Fig. 10.

**Figure 2:**
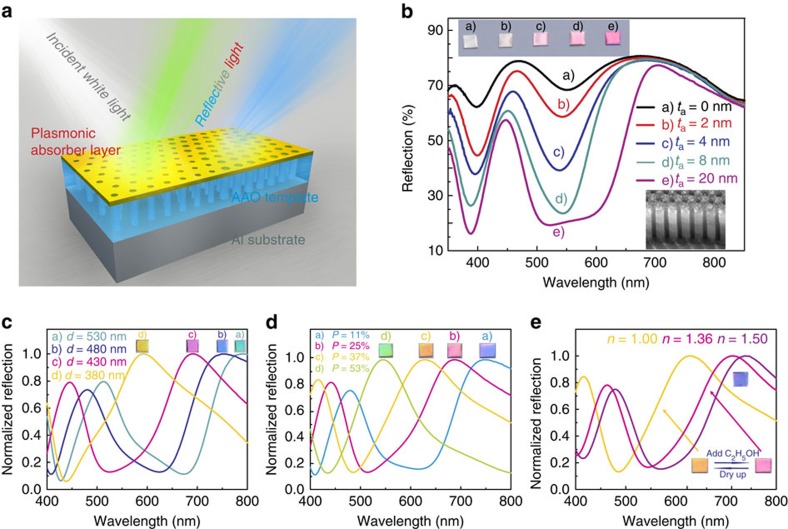
CTPM–PBA colour on porous structure. (**a**) 3D schematic overview of an AAO supported CTPM–PBA colour panel. The top layer is a PBA layer, the middle an AAO template, and the bottom an Al substrate. Light of specific colour is reflected in the case of white light incidence. (**b**) Measured reflective spectra of CTPM–PBA colour panels with different Ag sputtering time, *t*_*s*_. Top inserts are pictures of the corresponding samples. All the samples are about 4 × 4 mm^2^. The bottom-right insert is the SEM morphology of the sample e (*t*_*a*_=20 nm) observed at a tilted angle of 45°. Scale bar, 200 nm. (**c**) Reflective spectra of CTPM–PBA panels with different AAO thickness, *d*. Samples with longer *t*_g_ have larger *d*, resulting in redshift of reflective spectra. (**d**) Reflective spectra of CTPM–PBA panels (*d*=480 nm) with different porosity, *P*. Longer *t*_e_ result in bigger *P*, so as to small average refractive index, corresponding to reflective peak on the blue side. (**e**) Reflective spectra of CTPM–PBA panel (*d*=480 nm, *P*=37%) in different circumstances. Inserts in **c**, **d** and **e** are corresponding to camera pictures of the measured samples. All the samples are about 4 × 4 mm^2^.

As manifested above, using the CTPM–PBA approach, we successfully achieved large p–v ratios and narrow reflective peaks to realize the vivid colour display that is the basic of the full-colour plasmonic printing, which is lacking in previous colour-generation works based on the reflective metal–dielectric–metal (MDM) structures^28^,^29^,^30^. The reason lies in two notable advances made in our work: (i) we presented the CTPM to design the thicknesses of the dielectric layer and the top metal layer for high colour saturation by maximizing the reflective p–v ratio, while in previous works, the dielectric layer was designed as the Fabry–Perot resonator using the standing wave theory in which, at the targeted wavelength, the interference of the reflective wave at the inner interface of the thin metal layer is the DI, rather than the CI required by the CTPM. This condition of the CTPM is the reason for a remarkable blue-shift between the reflective valleys with the metal layer and without such layer (corresponding to the standing wave in dielectric layer) (Fig. 2b, Supplementary Fig. 4). In addition, the standing wave theory cannot guide the design of the top metal layer. (ii) More importantly, instead of the normal metal film in the MDM structures reported in previous works, the metal island film is introduced as the PBA to significantly sharpen the reflective peaks and give rise to a vivid colour output in the full-colour range, which is not feasible with previous MDM structures.

### CTPM–PBA colours on porous structure

After obtaining the ability to generate vivid colours, the CTPM–PBA structure can be further improved to develop a practical controllable plasmonic colour-printing approach with high functionality compatibility. To achieve this goal, we introduce an AAO template that takes the place of the solid dielectric layer (Fig. 2a), which can readily provide the potential for more controllable colour output and functional devices. As interpreted by Fig. 2b, a silver island film gradually forms with the increase in the average thickness of the Ag layer (*t*_a_; bottom inset in Fig. 2b), which remarkably enhances the p–v ratios in the reflection spectra, eventually generating vivid pink colour displayed on sample e (the result of a thicker silver island film is placed in Supplementary Fig. 10b and detailed comparing data of the p–v ratio can be found in Supplementary Table 1).

According to our theory of CTPM (Supplementary Note 2), the refractive index (*n*) and thickness (*d*) of the dielectric layer are key parameters in the modulation of the reflective spectra, so controlling *n* and *d* of the AAO template can be an effective method in modulating the appearing colour. In particular, since *n* is the average refractive index of the AAO layer, it is affected by three components that are the refractive index of Al_2_O_3_ in AAO film (*n*_A_=constant), the refractive index of the pores in the AAO film (*n*_P_), and the porosity of the AAO film (*P*, volume fraction of pores). Consequently, the output colour of our CTPM–PBA panel is mainly dominated by three key variable parameters, which are, *d* (ref. 31), *P*, and *n*_P_. To further explore the approach for output colour control, we attempted to modulate the colour of the CTPM–PBA panel by adjusting the parameters of *d*, *P* and *n*_P_ (Fig. 2). In Fig. 2c, the *d* of the AAO templates is controlled by changing the AAO growth time (*t*_g_). Values of *d* varying from 380 to 530 nm are readily obtained by choosing the *t*_g_ of 180, 220, 260 and 300 s, respectively (Supplementary Fig. 11), and correspondingly four colours from yellow to cyan are achieved. On the other hand, with an AAO that has fixed growing time, the colour can also be modulated by changing porosity of the AAO film, which can be realized facilely by etching the AAO film with acid. As illustrated in Fig. 2d, with the etching time (*t*_e_) adjusted from 0 to 75 min, the values of *P* ranges from 11 to 53% (Supplementary Fig. 12), which also contributes to a fruitful colour display. The pore's influence upon the metallic film's plasmonic properties is discussed in Supplementary Fig. 13.

Last but not the least, even without any changes to the structure of this CTPM–PBA panel, the colour of the CTPM–PBA panel can be effectively modulated. Due to the intrinsic characteristics of the porous structure, it is possible to fill the pores of the AAO with various dielectrics so as to tailor the *n*_*P*_, which enables controllable colour manipulation. In Fig. 2e, by changing the reflective index of the AAO pores from 1 (air) to 1.36 (ethanol) and finally to 1.50 (toluene), the displayed colour varies from yellow to blue. More interestingly, when the filled solution evaporates, the sample colour can reverse back to its original hue. This reversible colour transformation ability is known as chromotropic capability, which has been widely proved to be a valuable virtue for the application of information storage, decoration, camouflage and art^24^. Although its importance has been shown in many other fields, chromotropic properties are rarely performed in the field of plasmonic colour printing. More experimental observations regarding the relationship between the wavelength of the reflective peak and the refractive index of added dielectrics can be found in Supplementary Fig. 14.

### Full colour generation

Generally speaking, until now two obstacles have prevented plasmonic colour printing from achieving practical full colour generation. First, since most plasmonic colour printing approaches rely on the original surface plasmon resonance mode of the nanostructures, which are highly limited by various rigorous conditions, including structure geometry, the type of used metal, material crystallization and fabrication precision, it is hard to achieve realistic full-colour devices. Second, the conventional plasmonic colour-printing approaches typically involve complicated preparation equipment, such as EBL, which not only raise the fabrication difficulty and production costs, but also limit sample sizes. The existing traditional plasmonic printing methods cannot solve these problems simultaneously.

With the full understanding of the colour output origin of the CTPM–PBA panel, we found that the CTPM–PBA colour approach could serve as ideal solution for plasmonic full colour generation. Such colour generation is rooted in simple interference theory, so it is not difficult to predict reflective peaks or valleys, making the desired colours very accessible. Furthermore, the fabrication of CTPM–PBA colour panels is based on mature electrochemical method and sputtering techniques, both are known for their ease of production, scalable sample areas and low cost. Remarkably, it is feasible to achieve full-colour output by using aluminum, the third most abundant element on Earth, with costs at about 1000th as that of gold and silver, which is of great significance for device exploration^32^,^33^,^34^,^35^. It is also noteworthy that we found the island film of aluminum performs better as a PBA than silver (Supplementary Fig. 8). Moreover, the self-limiting impermeable oxide layer on the aluminum's surface can work as a protective coating, making aluminum-based devices more durable^36^.

**Figure 3:**
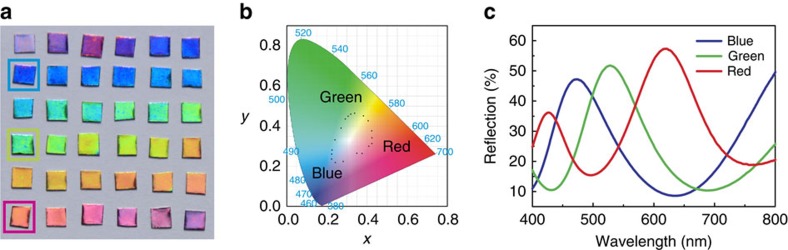
Full-colour Al CTPM–PBA palettes. (**a**) Camera picture of full-colour Al CTPM–PBA palettes. These palettes were achieved by implementing *t*_e_ differing from 0 to 138.5 min (Supplementary Table 2) on a CTPM–PBA panel with an original reflective peak at 692 nm under the incident angle of 8°. The samples are all about 3 × 3 mm^2^. To achieve these sub-pieces, the original AAO template is cut into sub-pieces by scissor before etching, which causes fluctuation of these samples that is then flattened using glasses. (**b**) Corresponding positions of part of samples in **a** plotted in the CIE 1931 colour space. The dots appeared as a circle, confirming the capability for achieving full colour using the CTPM–PBA panels. (**c**) Reflection spectra of the three samples marked with squares in **a**, displaying colours of blue, green and red, respectively.

The results from the CTPM–PBA palettes in full colour generation are shown in Fig. 3. A large piece of AAO template with a main reflective peak of about 692 nm at an incident angle of 8° was divided into 36 sub-pieces. The small pieces were then etched using diluted acid with different etching times to obtain different reflection spectra. Next, all the treated AAO templates were placed into the sputtering chamber. A layer of Al was sputtered onto them, bringing about colours ranging from red to blue. When plotting the positions of a portion of these colours in the CIE 1931 colour space, they appeared on a circle, confirming the realization of full colours in the Al CTPM–PBA palettes from the same original piece of AAO template (Fig. 3b). Taken as examples, the reflection spectra of the three sub-pieces of these palettes (marked in Fig. 3a) are shown in Fig. 3c, in which reflective peaks at 620, 528 and 472 nm correspond to red, green and blue colours, respectively. Also, we found some of the sub-pieces exhibited intensity changes in the colour display. This fact can be attributed to the damages caused by AAO template cutting, and can be effectively avoided in further applications that do not require such sample cutting.

### Colour printing with chromotropic functionalities

**Figure 4:**
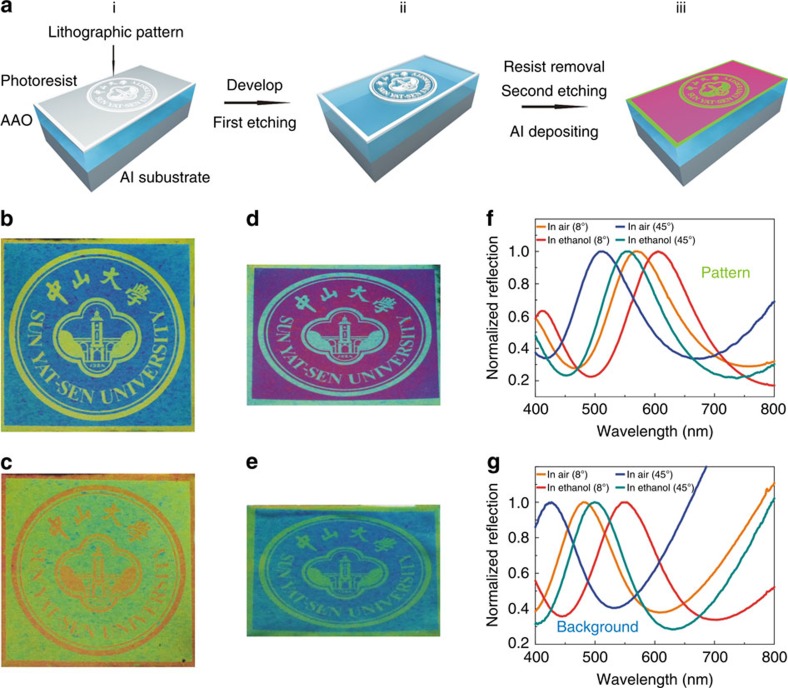
Colour pattern printing with chromotropic capability. (**a**) Method of colour pattern printing. (i) After spin-coated on an AAO template, the photoresist is patterned using photolithography. (ii) The photoresist is developed to leave the designed patterns, after which the first acid etching is implemented to form difference of effective thickness of the AAO template between patterned and unpatterned domain. (iii) An Al plasmonic layer is sputtered onto the sample to form the designed colourful pattern after the removal of photoresist and the implementation of the second etching. (**b**,**c**) Camera pictures of an as-prepared 15-mm-diameter badge of Sun Yat-sen University in (**b**) air and (**c**) ethanol, captured with a tilted angle of about 8°. (**d**,**e**) Camera pictures of the same badge in (**d**) air and (**e**) ethanol captured with a tilted angle of about 45°. (**f**,**g**) Reflection spectra of (**f**) the pattern and (**g**) the background of the badge in air or ethanol with the incident angles of 8° or 45°.

After achieving the ability to realize full-colour palettes using one original AAO template, we turned to the exploration of the Al CTPM–PBA technique for colour pattern generation, which plays a crucial role in practical colour printing. Unlike other plasmonic colour techniques that rely on very small structure elements, our CTPM–PBA colour approach can be far more efficient in printing large patterns when combined with photolithographic techniques, which makes it promising for scalable industrial applications. As a demonstration, a 15-mm diameter badge of Sun Yat-sen University was first printed onto a piece of Al slice using this approach. The badge was patterned in the positive-tone S1805 photoresist on a pre-prepared AAO template using ultraviolet direct-writing lithography, after which the AAO template was selectively etched and subsequently a layer of Al was deposited on the surface by sputtering, giving rise to a vivid colourful badge (Fig. 4a i-iii, experimental details can be found in Methods). The as-prepared sample is shown in Fig. 4b, with a yellow colour for the pattern and a cyan colour for the background. In this method, the direct-writing lithography used here can be replaced by mask lithography, which is more efficient and convenient for large pattern production. Also, the patterning process can be completed by hand drawing (good for art creation) or through 3D-printing technique (especially suitable for complex pictures) instead of lithography, as we demonstrated in the Supplementary Fig. 15. Thus, with such patterning techniques, the CTPM–PBA approach can be applied not only on ordinary colour pattern printing, but also on colouration and patterning for commercial or art products.

Due to the porous property of the AAO template, the CTPM–PBA panel can also exhibit chromotropic capabilities, paving the way for applications in functional colour devices. As demonstrated here, the colour of the as-prepared badge sample changed distinctly after moistened by ethanol, wherein the yellow colour turned orange for the pattern and the cyan colour became yellow–green for the background after the ethanol filled the pores of the AAO template (Fig. 4c). This process is reversible, that is, the colours will revert to the original hues after the badge has dried. This chromotropic colour printing is applicable for anticounterfeiting of trademark, where the colours of patterns on the CTPM–PBA panel can turn into other specific colours when moistened by liquid with selected refractive index.

Moreover, as shown in Fig. 4d, the colours of a CTPM–PBA panel can also change by adjusting the observing angle; this angle-dependent property can be used to further enrich the functionalities of the CTPM–PBA colour approach when combined with the chromotropic characteristic, where more colour combinations can appear on a completed CTPM–PBA panel. For instance, a new combination of colours appeared after the ethanol-moistened badge was observed at a tilted angle of 45°, where the pattern was yellow–green and the background was cyan (Fig. 4e). With this enhanced chromotropic capability, which provides a two-dimensional colour-modulating approach (refractive index and observed angle) and more colour combinations in a pattern on a completed CTPM–PBA panel, the CTPM–PBA colour approach will have better performance in anticounterfeiting as well as special labelling, functional filters and even the visual arts. The corresponding reflection spectra of colour-tuning performance are presented in Fig. 4f,g. The relationship between the displayed colours and the observing angles is detailed discussed in Supplementary Fig. 16.

## Discussion

According to above analyses, one of the essential advantages of our proposed CTPM–PBA colour approach is its simple origin, which makes the design of colour very accessible, enabling full-colour generation by tailoring the locations of reflective peaks by the method of etching an AAO template with acid. By fitting the experimental data, we were able to obtain a linear approximation of the relationship between the reflective peak and etching time in experiment, which can be written as


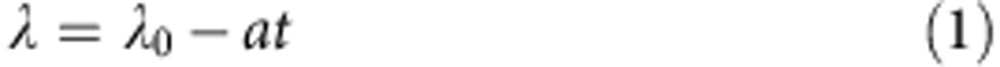


where *λ* is the reflective peak wavelength of the final displayed colour on the CTPM–PBA panel, *λ*_0_ the original reflective peak wavelength of the AAO template before etching, *t* the etching time, *a* the coefficient describing the decay rate of peak wavelengths against etching times. For example, for the samples presented in Fig. 3, where *λ*_0_ is 692 nm, *a* is fitted to be about 2 nm min^−1^ (the fitting data can be found in Supplementary Fig. 17). With this linear relationship, the colour design for subsequent device is easily done, making this CTPM–PBA colour technique suitable for real-world industrial applications.

**Figure 5:**
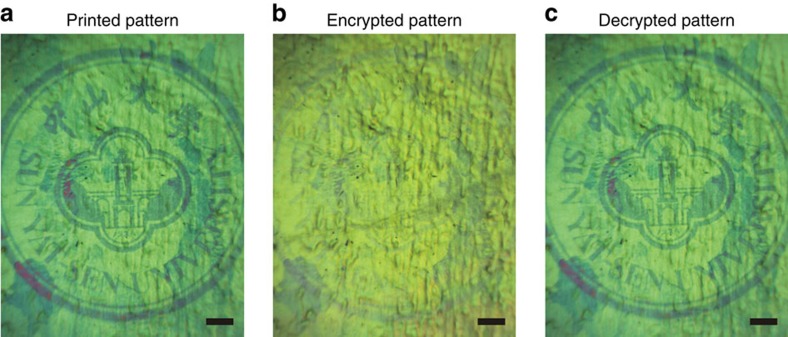
High-density information encryption storage. (**a**) A colourful 500-μm-radius badge of Sun Yat-sen University printed on a CTPM–PBA panel. (**b**) The same badge after spin coated with a layer of PMMA. The displayed colours came into another colour domain (green), and the difference between the pattern and the background became blurry. (**c**) Clear pattern of the badge after the PMMA layer was removed. Scale bar, 100 μm.

As was pointed out in the introduction, there are three key problems that hinder the development of plasmonic colour printing: full-colour generation, scalable fabrication and device functionalization. On the basis of above demonstrations, it is evident our CTPM–PBA approach has shown full colour output ability, and as a plasmonic printing method without the need for EBL it is practical for applications that can easily fabricate plasmonic printing products with areas up to the scale of a centimetre. In addition to these two important advances, the applications of our CTPM–PBA colour technique can also be further explored to develop novel plasmonic printing devices with multi-functionality. As an interesting demonstration, we have discovered a capability to hide information with the use of our chromotropic CTPM–PBA colour approach. Moreover, since this functionality can be achieved at a very small scale with pixel size ∼1 μm (Supplementary Fig. 18), our presenting approach shows a promising future in high-density data encryption storage and related fields (Fig. 5). A 500-μm-radius badge of Sun Yat-sen University was printed in a CTPM–PBA colour panel using the method exhibited in Fig. 4a, showing a blue–green colour pattern distinctly differing from the green colour in the background (Fig. 5a). We carefully chose the blue–green colour regions, where colour has a high changing rate against wavelengths, so it is possible to distinguish two colours with a small wavelength difference in the spectra. Subsequently, after the panel was coated with a layer of poly(methyl methacrylate) (PMMA), a well-known transparent material, the refractive index in the pores of the AAO template was changed, resulting in a colour redshift of our sample, both in the pattern and background areas. Since the shifted colours were both located in the green range, the pattern was indistinguishable from the background, which renders the recorded information invisible (Fig. 5b). When the PMMA was removed, the original pattern appeared again (Fig. 5c). The PMMA not only is a part of our method, but also serves as a protective layer for preventing surface damage. This information hiding technique is also compatible with the large size patterning approach with two cycles of photolithography and acid etching prior to plasmonic layer deposition, enabling high-density encrypted data hided in large colour patterns, which could be very promising in security-related applications.

In conclusion, we introduced a CTPM–PBA approach into the field of plasmonic colour printing. This approach can be significant to the development of practical plasmonic devices due to its characteristics both in the simple origins of colour design and in the easy implementation of sample manufacturing. Using the CTPM–PBA colour approach, we have been able to solve the three key problems in the field of plasmonic printing, readily achieving full-colour generation on large sample areas up to the scale of a centimetre, which is the largest scale ever reported in the field of plasmonic colour printing. Furthermore, the chromotropic characteristic of this colour approach has been investigated in depth, demonstrating that our CTPM–PBA colour approach could play an important role in various functional printings, such as anticounterfeiting, special labels, and high-density data encryption storage. Our work not only developed new theory and concept in plasmonic colour printing, but also presented a practical approach for converting the potentials of plasmonic colour printing into realistic applications, which could enrich our colourful world in an innovative way.

## Methods

### Optical and morphology characterization

All reflection spectra were measured on an ultraviolet/visible/near-infrared spectrometer (Lambda 950, PerkinElmer) at an incident angle of 8°. The images of the small patterns in Fig. 5 were observed and captured using an upright reflective microscope (Olympus MX51, Olympus Inc.) equipped with a digital camera (DCC1645C, Thorlabs). Other camera pictures were captured using a Canon EOS 7D camera. The SEM images were taken by a Zeiss Auriga-39-34 (Oberkchen, Germany) microscope operating at 5.0 kV.

### Numerical simulations

The simulation results shown in Fig. 1d were calculated with Finite-difference Time-domain (FDTD) method^37^, using a commercially available FDTD simulation software package from Lumerical Solutions. The average value and root-mean-square (r.m.s.) of the rough silver layer's thickness were both set as 8 nm. The corr lengths (average correlation lengths of roughness) are set as 10 × 10 nm. The permittivity of silver was taken from Palik^38^. The used refractive index for the Al_2_O_3_ was 1.76.

### SiO_2_ film deposition

The SiO_2_ film was deposited using ICP-PECVD (PlasmaPro System 100, Oxford) under 75 °C. The flow rate of N_2_O and SiH_4_ are 13.0 and 4.0 s.c.c.m., respectively.

### Fabrication of CTPM–PBA panel

The AAO templates were fabricated by a two-step anodization process^39^,^40^,^41^,^42^. Pure aluminum sheets (99.999%) were electropolished in a mixture of HClO_4_ and C_2_H_5_OH with the volumetric ratio of 1:3 to smooth surface, at a constant current of 1.2A and temperature of 0 °C. Then the aluminum sheets were anodized in 0.3 M oxalic acid with a constant voltage of 45 V and temperature of about 4 °C for 3 h to form the first AAO layer, which were removed in a mixture of 6 wt% phosphoric acid and 1.8 wt% chromic acid at 60 °C for 1.5 h in the followed step. Next, regular AAO templates were produced by anodization under the same conditions as the first anodization, but with anodizing duration at the scale of 100 s. Since the thickness of as-prepared AAO template can monotonically increase with the increase in anodization time, and the pore diameter and period have linear relationship with the applied oxidation voltage (40 and 110 nm in diameter and period, respectively, for the oxidation voltage of 45 V), the thickness and porosity of as-prepared AAO template can be controlled by anodization time and voltage. Moreover, we used oxalic acid to etch the as-prepared samples, by which we could further modulate pore size and distance by choosing different etch time. For the as-prepared AAO template with oxidation voltage of 45 V, we found the porosity of AAO template (*P*) and the etch time *t*_e_ follows the relationship of: *P* (%)=0.0069(40+0.65 *t*_e_)^2^, where the unit of *t*_e_ is min.

To achieve a predesigned colour with an as-prepared AAO template, the template is etched in 0.3 M oxalic acid at a temperature of 45 °C for a proper duration. Afterward a layer of plasmonic layer is coated onto the as-treated AAO template in a Quorum Q150T ES sputtering system, so as to form the vivid colour of a CTPM–PBA panel. A silver layer was coated under a sputter current of 3 mA with proper sputter time mentioned above, while aluminum layer coated under a sputter current of 100 mA and sputter time of 100 s (thickness of sputtered Ag and Al layer is discussed in Supplementary Fig. 19).

### Colour pattern printing process

A layer of positive-tone S1805 photoresist was spin-coated onto a fresh AAO template and then predesigned pattern was printed in the photoresist using direct write photolithography on a Heidelberg uPG501 system. The sample was subsequently developed in 2.38% TMAH to form a relief pattern in photoresist, enabling selective etching under the same condition described above with duration of 40 min. After the first etching, the remaining photoresist was removed by acetone at 60 °C for 1 h to expose all the surface of the AAO template, followed by a second etching with duration of 40 min. Then an aluminum layer was coated on the as-treated AAO template, giving rise to two vivid colours to domains of designed pattern and background, respectively.

### Implementation of high-density data encryption

First, patterns carrying information in small scale were printed onto a CTPM–PBA colour panel using method described above, forming colourful ‘data' which can only be clearly seen in a microscope. Afterward, a layer of PMMA (molecular weight: 350,000, 5 wt% in chlorobenzene) was spin-coated onto the as-edited CTPM–PBA panel at 4,000 r.p.m. for 35 s. The PMMA layer functioned as an encryption component to hide the printed data from the background. When decrypting the encrypted data, this PMMA layer was dissolved in chlorobenzene under a soft ultrasonic process sustaining 8 min, after which the encrypted data became apparent again. As for the sample in Fig. 5, the duration of first etching is 2.5 min and that of second etching is 40 min.

## Additional information

**How to cite this article:** Xue, J. *et al.* Scalable, full-colour and controllable chromotropic plasmonic printing. *Nat. Commun.* 6:8906 doi: 10.1038/ncomms9906 (2015).

## Supplementary Material

Supplementary Information Supplementary Figures 1-19, Supplementary Tables 1-2, Supplementary Notes 1-2 and Supplementary References.
