# Supplementary material for: Scalable, full-colour and controllable chromotropic plasmonic printing
Source: Nat Commun. 2015 Nov 16;6:8906. doi: 10.1038/ncomms9906 (PMC4660354; doi:10.1038/ncomms9906)
Supplement: Supplementary Information — Supplementary Figures 1-19, Supplementary Tables 1-2, Supplementary Notes 1-2 and Supplementary References. [file ncomms9906-s1.pdf]

Supplementary Figures

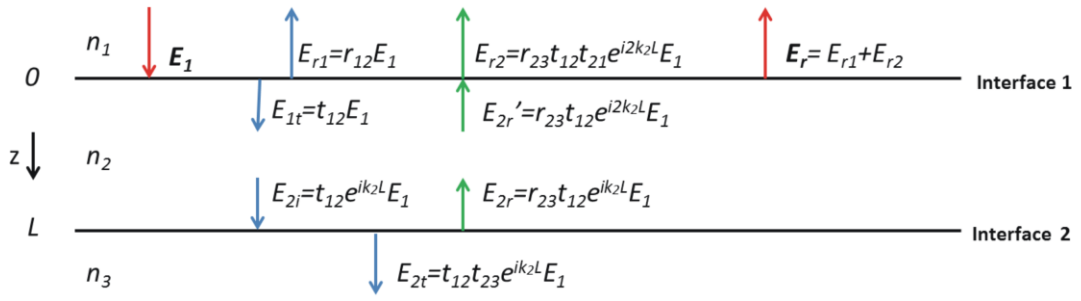

Supplementary Figure 1 Reflective and refractive behaviors of light with normal incidence in a three layer system. E_1 and E_r are the complex amplitudes of the incident wave and final reflected wave, respectively. The parameter r_{ab} and t_{ab} are corresponding reflection and transmission coefficients, respectively. Detailed discussion is placed in Supplementary Note 1.

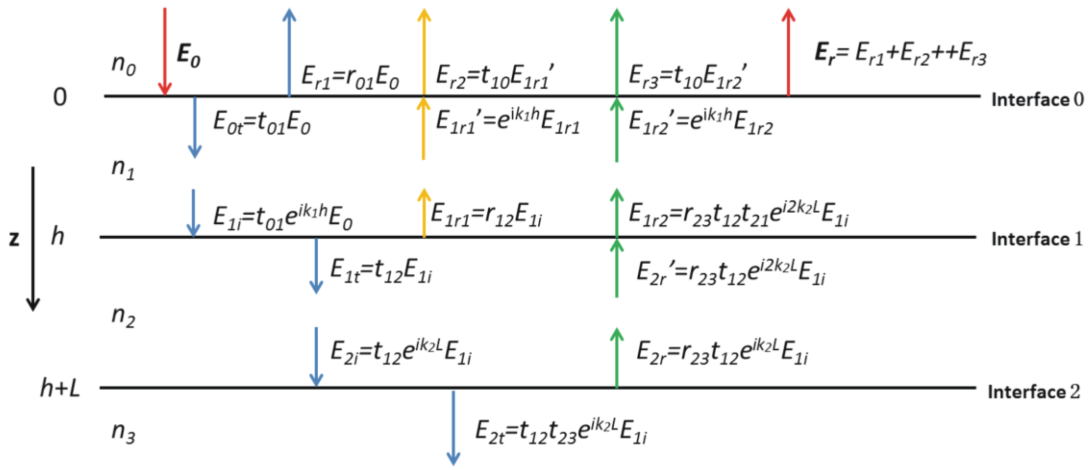

Supplementary Figure 2 Reflective and refractive behaviors of light with normal incidence in a four layer system. E_0 and E_r are the complex amplitudes of the incident wave and final reflected wave, respectively. Detailed discussion is placed in Supplementary Note 2.

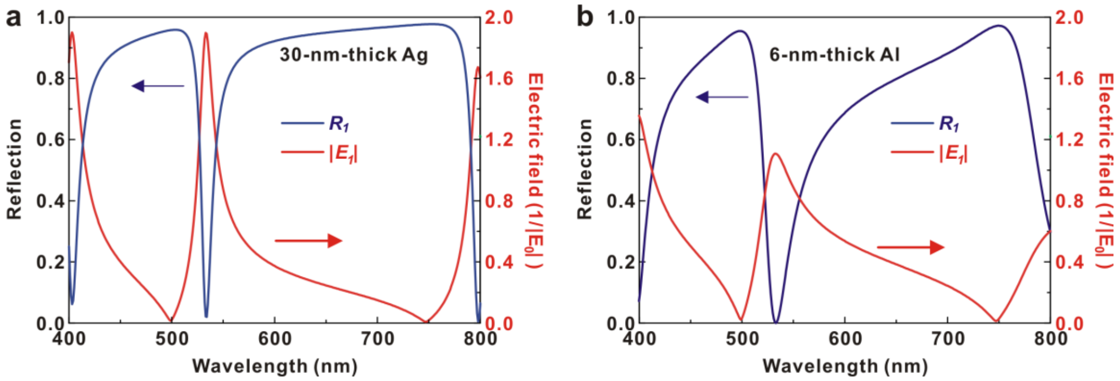

Supplementary Figure 3 Reflection spectra of the structure and the electric field intensity in the metal layer calculated for a 400-nm-thick Al_2O_3 with different metallic coating. (**a**) 30-nm-thick Ag. (**b**) 6-nm-thick Al. The maximum of E_1 represents CI in the metal layer, while the minimum of reflection (R) represents the DI of the reflected light waves. It is easy to find that the conjugate twin phase modulation with an ultrathin plain metal layer can distinctly enlarge the reflective p-v ratio. The substrate is set to be silver. The refractive index of Al_2O_3 was set as 1.76, and that of Ag and Al were taken from Palik[3], which is the same in all parts of this Supplementary information.

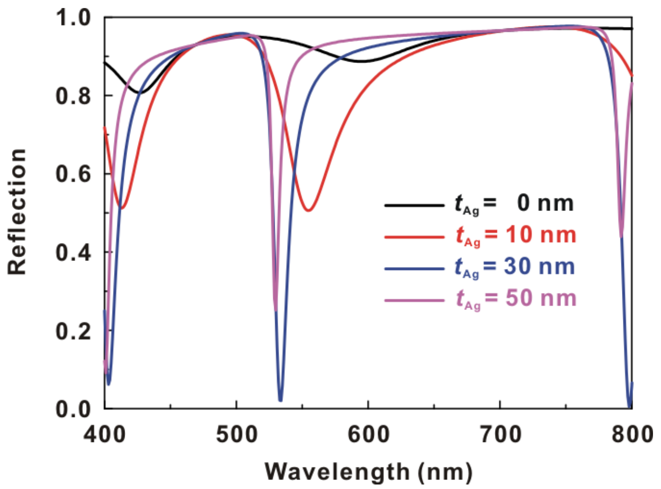

Supplementary Figure 4 Calculated reflection spectra calculated for a 400-nm-thick Al_2O_3 with metallic coating of different thickness. With the increase of the thickness of Ag coating (t_{Ag}), the wavelengths of valleys have blueshift and p-v ratio increase at first but decrease for too thick Ag coating (thicker than 30 nm). It is worth noting that reflective valley has obvious blue-shift after the coating of silver coating, the reason of which is illustrated as follow. Before the metal coating, reflective valley corresponds to destructive interference (DI) of the reflective wave at the top of dielectric layer. After the metal coating, the interference mode will be changed by the participation of the metal layer. In this case, distinct reflective valley requires constructive interference (CI) occurs at the top of the dielectric layer to arouse maximum e-field and maximum absorption in the metal layer. Because of the non-trivial optical phase transition caused by the metal-dielectric interface, the wavelength of the new CI mode is located at the blue side of the wavelength of the original DI mode without metal coating, so the reflective valley blueshifts after the metal coating. This result can also be found in Fig. 1b and c, where the wavelength of $\Delta\varphi_{21} = 2\pi$ (CI) in Fig. 1c is at the blue side of that of $\Delta\varphi_{21} = \pi$ (DI) in Fig. 1b.

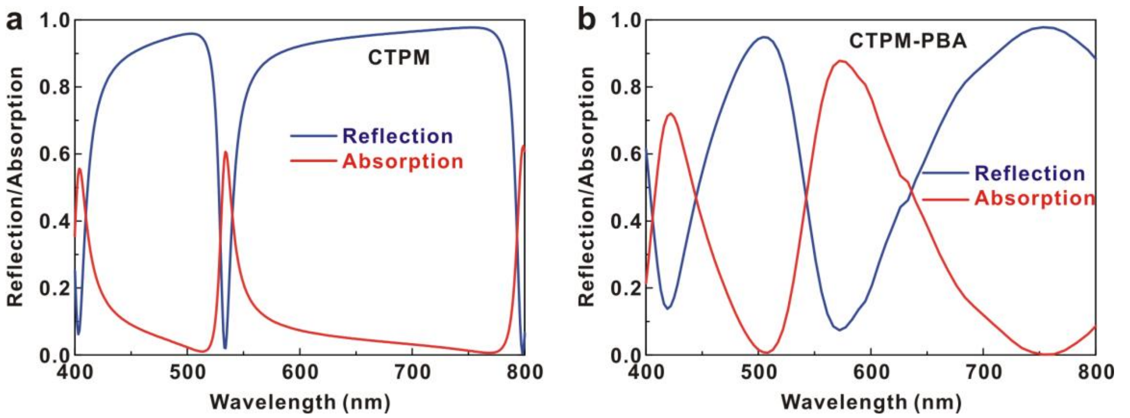

Supplementary Figure 5 Reflection spectra of the structure and absorption of the metallic layer calculated for a 400-nm-thick Al_2O_3 with different silver coating. (**a**) 30-nm-thick plain Ag layer. Enhanced light absorption only appears in a very limited range, resulting in narrow reflective valleys but wide and flat reflective peaks. (**b**) Rough Ag layer with average thickness of 8 nm. Wide spectral range of light absorption is created, making the reflective peaks much narrower. The substrate is silver. The results in (b) are obtained by the simulation in FDTD solutions.

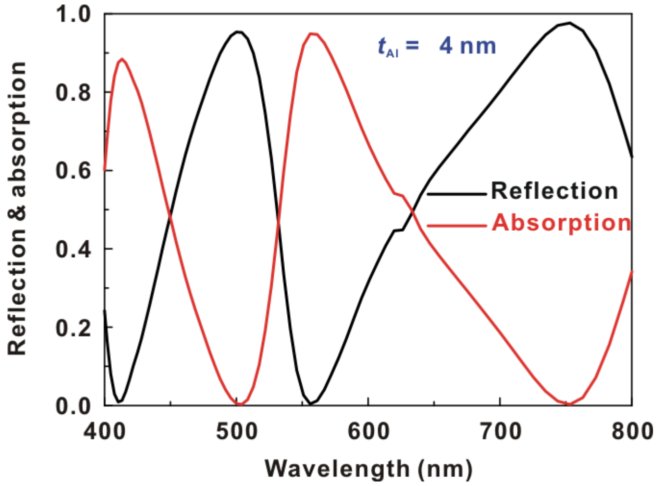

Supplementary Figure 6 Reflection spectra of the structure and the Absorption of the plasmonic layer simulated for a 400-nm-thick Al_2O_3 with Al island film. The average thickness of Al island film (t_{Al}) is 4 nm. These results are simulated using FDTD solutions.

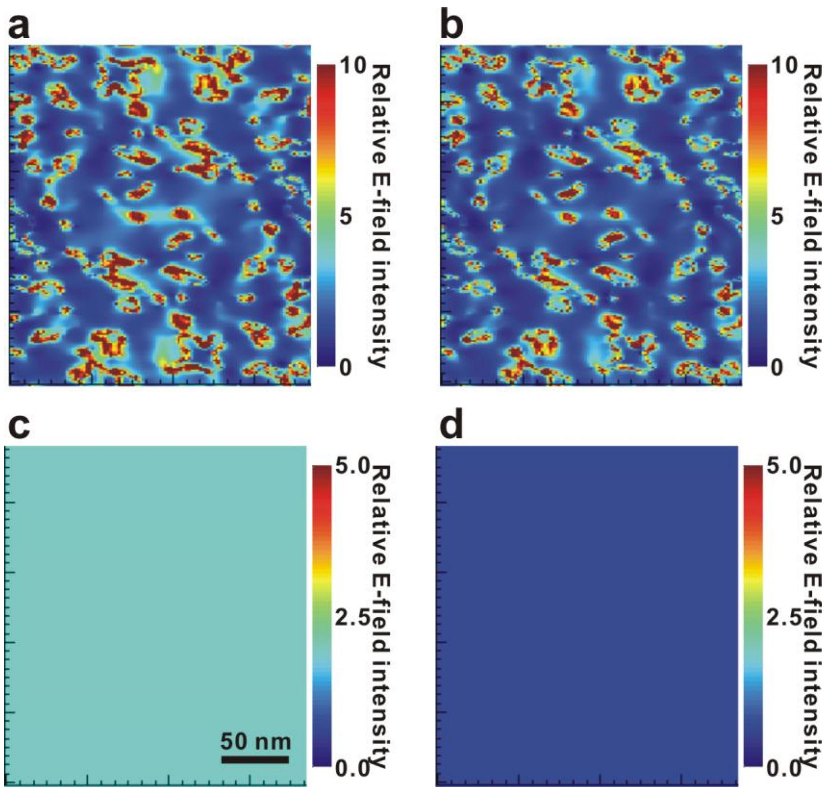

Supplementary Figure 7 Simulated electric field distributions at bottom surface of the metallic layer. These results were calculated at the wavelengths of (**a**) 572 nm (reflected valley) and (**b**) 552 nm in the case of rough silver layer, and (**c**) 531 nm (reflected valley) and (**d**) 522 nm in the case of plain silver layer. Strong E field enhancement still exists at the wavelength 20 nm away from the reflective valley under CTPM-PBA structure, unlike the case of plain metal coating where the electric field already becomes very weak at the wavelength only 10 nm away from the reflected valley, revealing the broadband electric field enhancing effect of the rough silver island film. The simulated structures are accordant to the description in Supplementary Fig. 3.

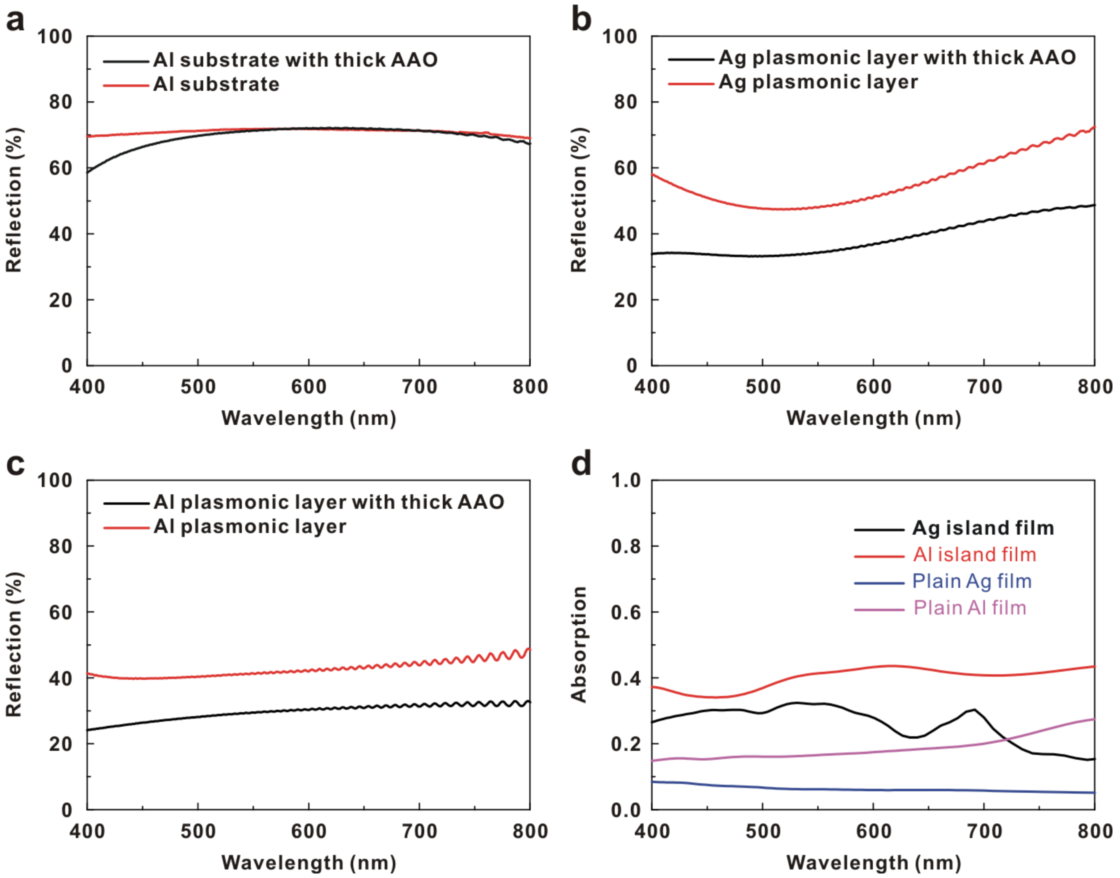

Supplementary Figure 8 Optical properties of the plasmonic layer. (**a**) Reflection spectra of Al substrate without AAO (red curve) and with thick AAO (black curve). The plain profile of the red curve, measured after the removal of AAO, indicates the role of Al substrate as an unselective reflector. The black curve indicates that thick AAO doesn't support obvious peak in the visible range and has only slight light absorption. (**b**) Reflection spectra of Ag plasmonic layer on a thick AAO with Al substrate (black curve) and after divided by the reflection of Al substrate with thick AAO (red curve). The red curve represents the absorption characteristic of the Ag plasmonic layer, indicating the property of broadband absorber and slightly preferential absorption around the wavelength of 520 nm. The average thickness of Ag is about 8 nm. (**c**) Reflection spectra of Al plasmonic layer on a thick AAO with Al substrate (black curve) and after divided by the reflection of Al substrate with thick

AAO (red curve). The plain profiles of the spectra indicated Al plasmonic layer is an excellent broadband absorber in the visible range. The average thickness of Al layer is about 50 nm. Reflection spectra in (a), (b) and (c) were measured under an incident angle of 8°. (**d**) Simulated Absorption of metallic island film and plain metal film. The average thickness of Ag and Al island film and thickness of plain Ag and Al film are all 8 nm. The simulated Absorption spectra indicate the high absorbing efficiency and broadband property of the metallic island film. As demonstrated in the SEM pictures in the inserts of Fig. 1e and Fig. 2b, the metal island film consists of nano-particles with random size and shapes, which lead to the origin of the broadband absorption. The different metal nano-particles have different plasmonic resonant wavelengths, which form the broadband absorption. Furthermore, the diffusion scattering of the light caused by these particles may increase the optical path of light in the metal layer, and then enhance the intensity of absorption. It is worth noting that the more flat spectra of aluminum island film demonstrate the better performance of aluminum than silver to be plasmonic broadband absorber.

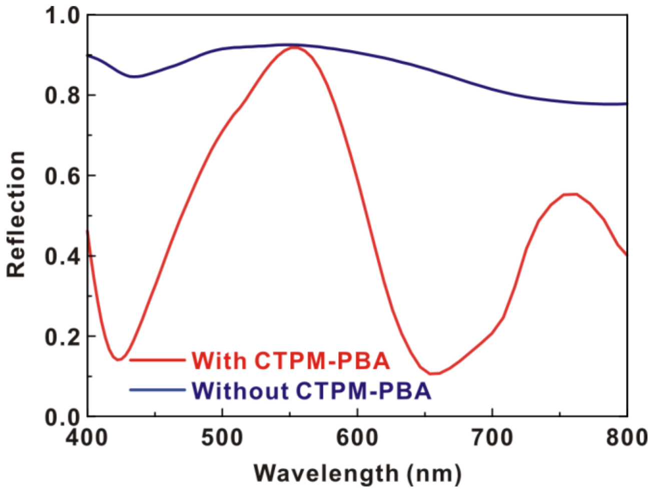

Supplementary Figure 9 CTPM-PBA effect on other dielectric and substrate. This figure shows simulated reflection spectra of the aluminum supported 360-nm-thick SiO_2 thin film without (blue curve) and with (red curve) the coating layer of a rough silver island film (average thick equals to 8 nm), revealing that the CTPM-PBA effect is independent to the type of dielectric layer film and substate. The refractive index of SiO_2 is set as 1.46.

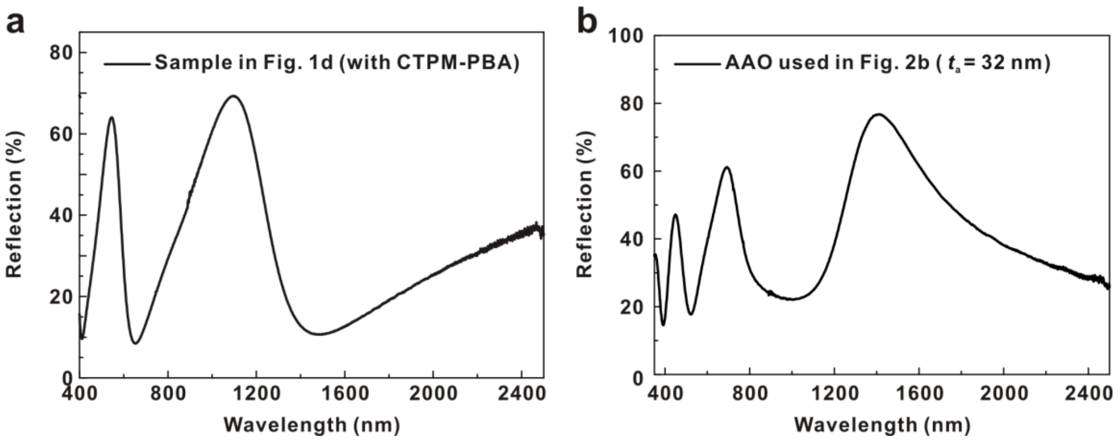

Supplementary Figure 10 Reflection spectra showing the order of the reflective peaks. (**a**) Sample in Fig. 1d (with CTPM-PBA). (**b**) AAO used in Fig. 2b coated by a silver island film with the average thickness of 32 nm. The spectra were measured under an incident angle of 8°. As shown in these figures, the main peak in Fig. 1d is related to 2nd order, and those in Fig. 2b are related to 2nd and 3rd order. According to these results and the thickness of the samples in our experiments, the main peaks in Fig. 3 and 4 are related to 2nd order, and those in Fig. 2c are related to 2nd and 3rd order.

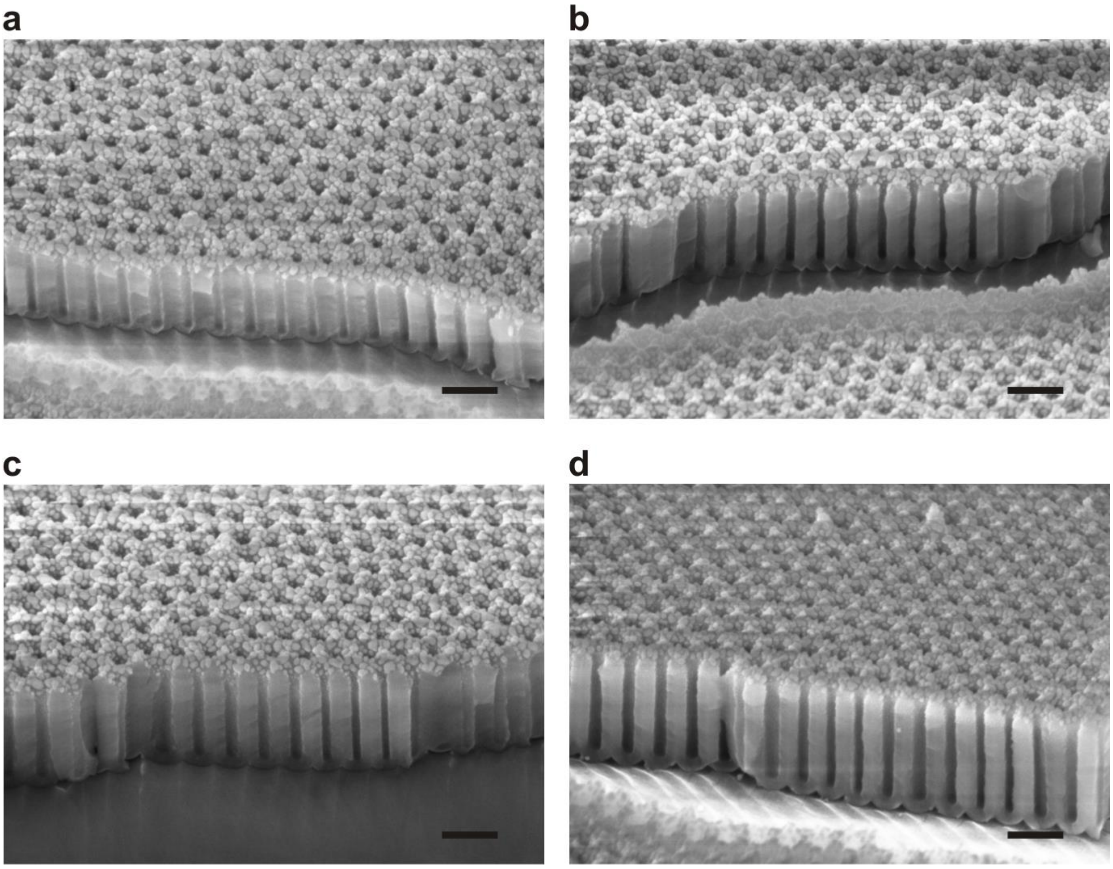

Supplementary Figure 11 SEM images of AAO template with different growth time, t_g. (**a**) t_g = 180 s. (**b**) t_g = 220 s. (**c**) t_g = 260 s. (**d**) t_g = 300 s. These samples were observed at a tilted angle of 45°. Scale bar, 200 nm.

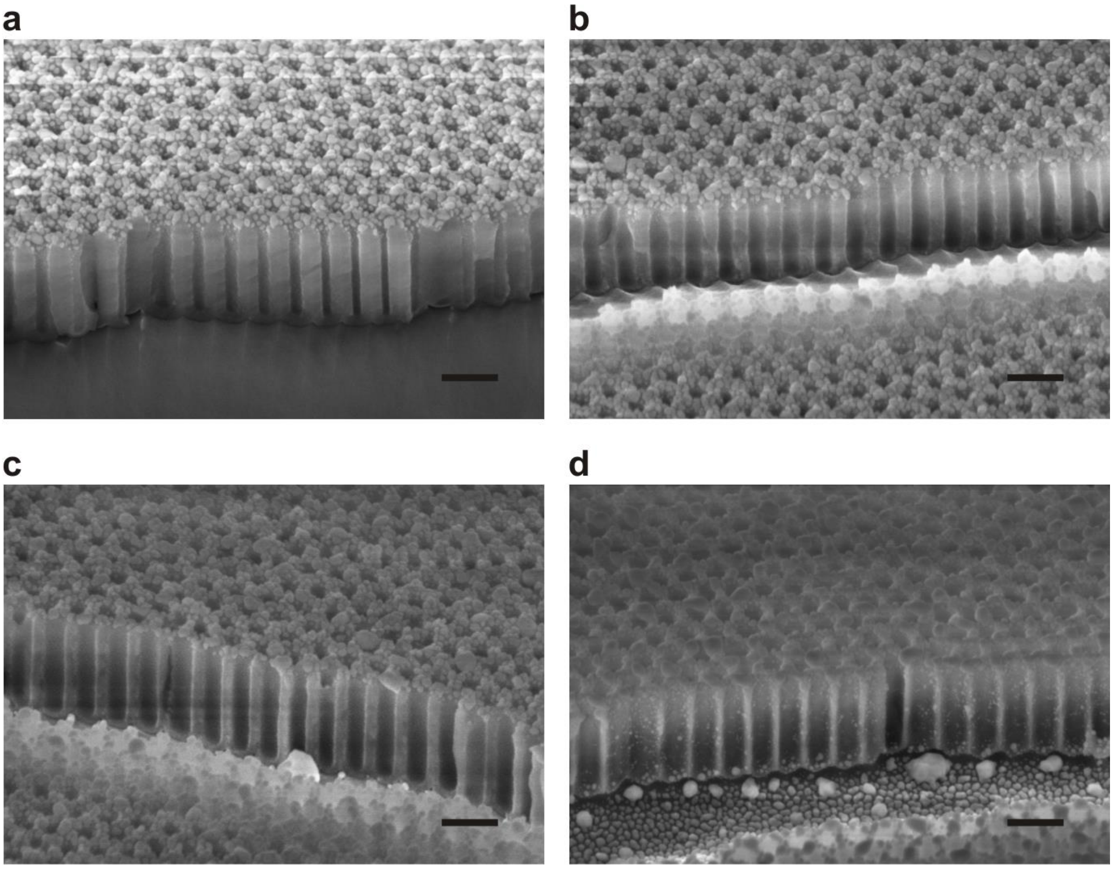

Supplementary Figure 12 SEM images of AAO template (t_g = 260 s) with different etching time. (**a**) t_e = 0 min. (**b**) t_e = 25 min. (**c**) t_e = 50 min. (**d**) t_e = 75 min. These samples were observed at a tilted angle of about 45°. Scale bar, 200 nm.

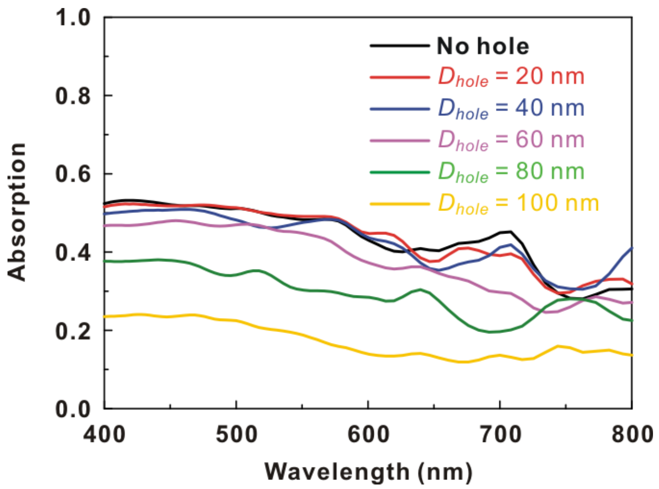

Supplementary Figure 13 Simulated absorption of the pure Ag island film with holes of different diameter (D_{hole}). The holes are hexagonally arranged and have the period of 110 nm. As shown by the data, the specific absorbing intensity is influenced by the hole size, but the Ag island film still maintain the property of broadband absorption.

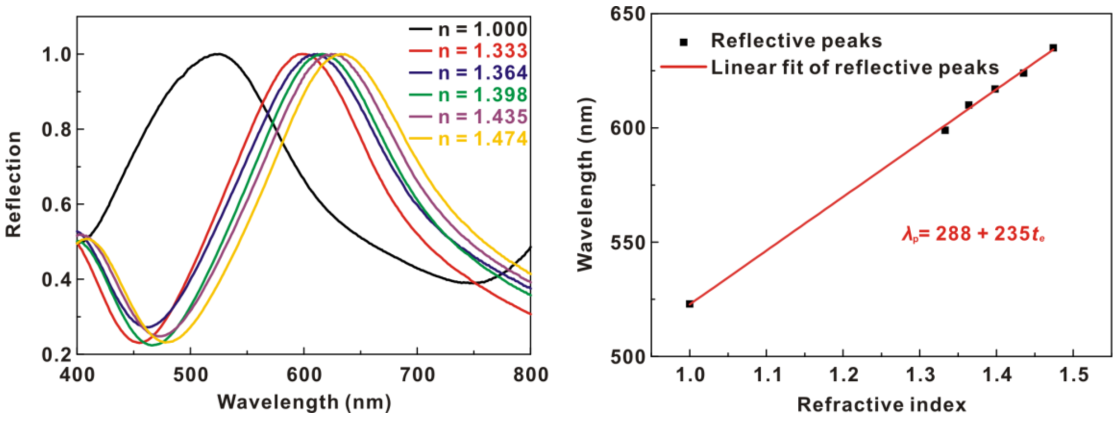

Supplementary Figure 14 Experimental observation of relationship between the reflective peak's positions and the refractive index of the pores in the CTPM-PBA panel (t_g = 220 s, t_e = 75 min). (**a**) The reflective spectra of the CTPM-PBA panel in glycerine solution (in water) with different concentrations of 0 wt%, 25 wt%, 50 wt%, 75 wt% and 100 wt%, corresponding to the refractive indices of 1.000, 1.333, 1.364, 1.398, 1.435 and 1.474, respectively[4]. (**b**) The linear fit result of the relationship between the reflective peak wavelengths (unit is nanometer) and the refractive index of pores in the CTPM-PBA panel, showing that this relationship is very linear.

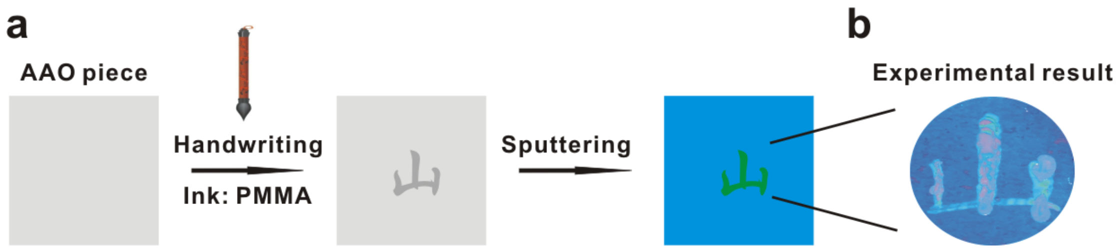

Supplementary Figure 15 CTPM-PBA colour pattern with handwriting. (**a**) Patterning process with handwriting. After a pattern is drawn by pen using PMMA solution as ink, a metallic layer is sputtered on the sample to form colourful patterns. (**b**) The experimental demonstration of CTPM-PBA colour pattern (a Chinese character) by handwriting. The concentration of PMMA solution is 1.5 %wt. The sputtered metallic layer is a silver plasmonic film with average thickness of about 12 nm. The handwriting process can also be replaced by 3D-printing technique, which has great advantages in generating complex patters and can precisely control the output liquid flux, so as to form all kinds of designed colourful figures.

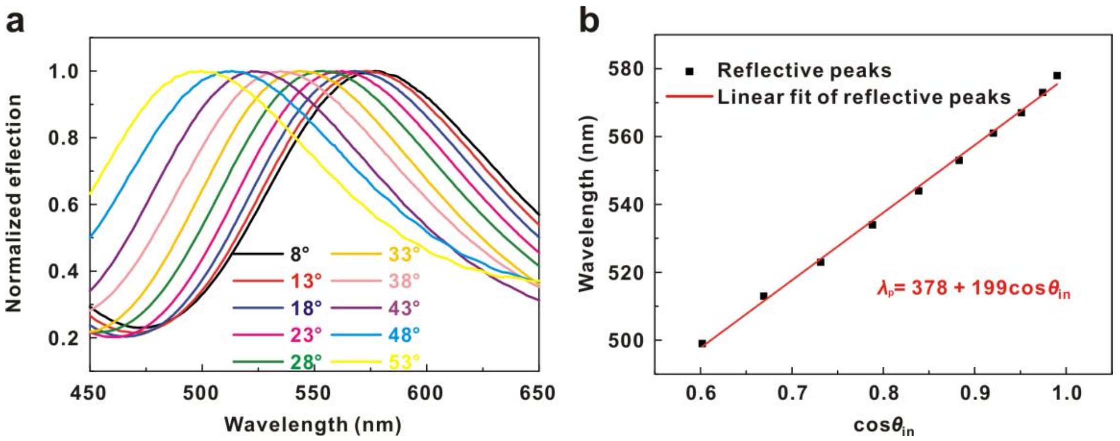

Supplementary Figure 16 The relationship between the wavelength of reflective peak and the incident angle. (**a**) The reflection spectra of the pattern in Fig. 4 with the increase of incident angle. (**b**) Linear fit of the wavelengths of reflective peaks against the value of $cos\theta_{in}$. The results indicate the linear relation between reflective peak and $cos\theta_{in}$.

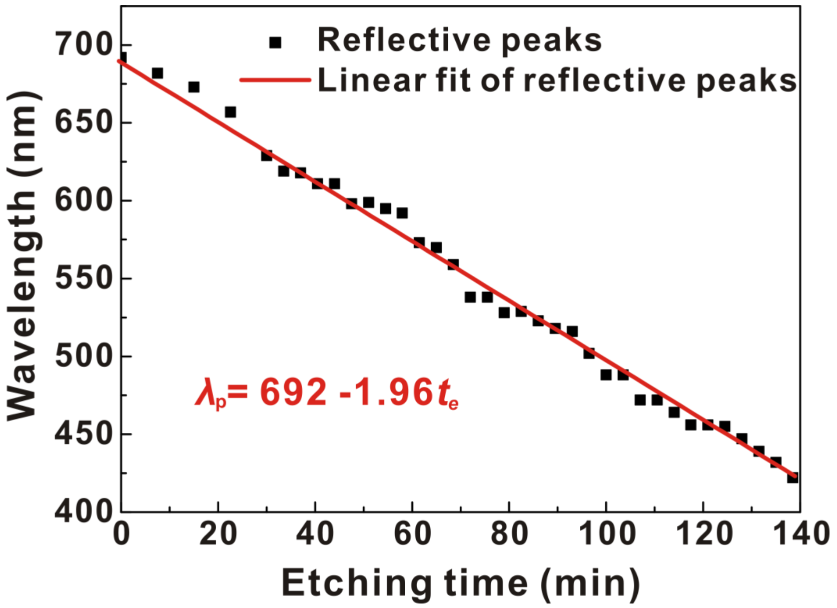

Supplementary Figure 17 Linear fitting between reflective peak wavelengths and etching time. The red curve fits well with the dots of peaks against the etching time, indicating that there is a linear approximation of the relationship between reflective peak wavelength and etching time, which can be written as λ_p = 692 -1.96t_e, where the unit of wavelength is nanometer and that of time is minute. The data of the peaks were obtained from the samples displayed in Fig. 3a.

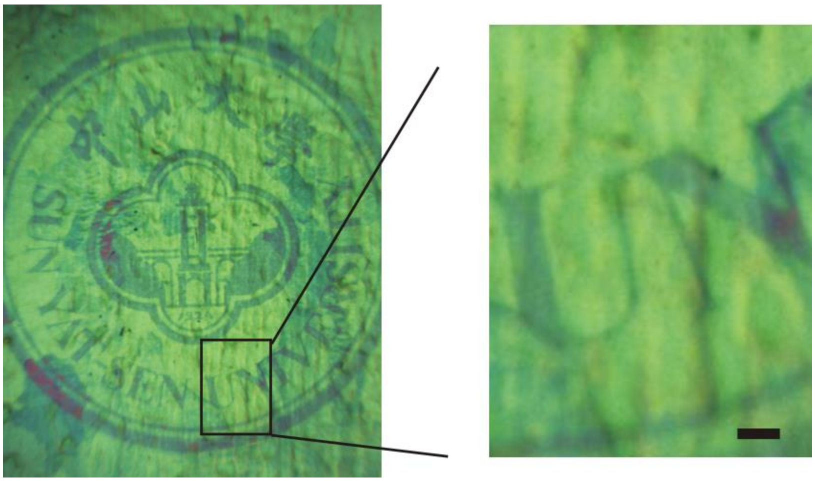

Supplementary Figure 18 Narrow line displayed on the CTPM-PBA panel. Scale bar, 20 μm. Narrow line with linewidth about 4 μm can be clearly displayed in the magnified picture, proving that the pixel size in our experiments is small than 4 μm. One the other hand, the focus laser size in the process of photolithography is about 1μm. So the pixel size in our experiments is between 1 and 4 μm. Moreover, since the pore period of AAO is only about 110 nm, we believe this CTPM-PBA technique possesses great potential in reaching pixel size at the scale of diffraction limit when combined with other lithograph approach of higher precision.

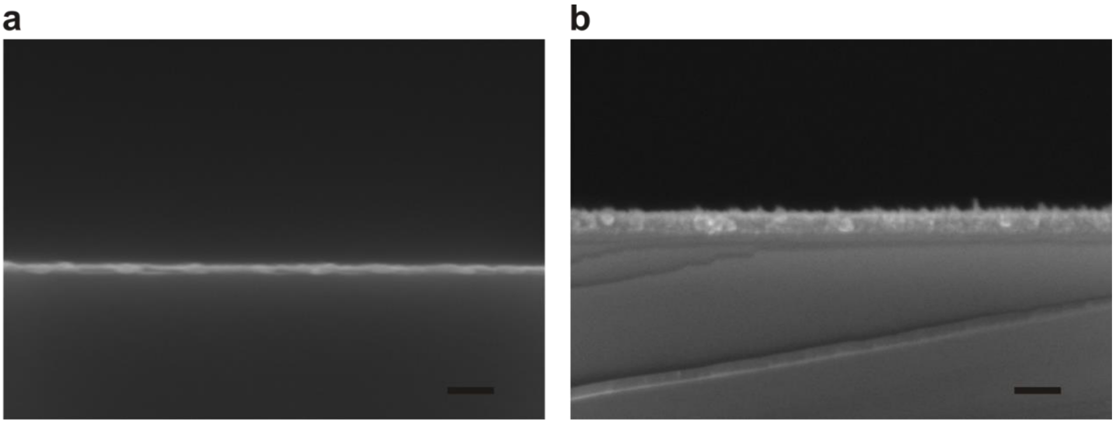

Supplementary Figure 19 SEM cross-section images of sputtered Ag and Al layer. (**a**) A silver layer sputtered on a silicon wafer with the sputter current of 3 mA and the duration of 250 s. (**b**) An Al layer sputtered on a silicon wafer with the sputter current of 100 mA and the duration of 100 s. Scale bar, 100 nm. The as-prepared Ag layer thickness is about 20 nm, and the Al layer thickness is about 50 nm. These thicknesses of the plain metal layer on silicon can be regard as the average thickness of the metallic island films fabricated on our CTPM-PBA panels under the same sputtering conditions. We note that the thicknesses of Al and Ag are quite thick. The main reason for this fact is that our sputtering procedure also accompanies with the process of oxidation, which makes the deposition layer impure. So, in order to serve as broadband absorber layer, the impure metal layer needs to have relatively larger thickness than theoretical results. Fortunately, also due to the impurity, the experimentally prepared metallic broadband absorber does not block most of the light, as proved by Fig. 3c in main text, where the reflection over 50% and high p-v ratio can be found.

.

Supplementary Tables

Supplementary Table 1 Change of peak-valley ratios of reflection spectra with increasing thickness of metal layer.

t_a (nm)	Valley 1	Peak 1	Valley 2	Peak 2	p-v ratio 1	p-v ratio 2
0 s	0.62	0.79	0.68	0.81	1.27	1.19
2	0.45	0.75	0.59	0.80	1.67	1.36
4	0.38	0.68	0.39	0.79	1.79	2.03
8	0.26	0.61	0.24	0.79	2.35	3.29
20	0.16	0.57	0.19	0.77	3.56	4.05
32	0.15	0.47	0.18	0.61	3.13	3.39

Valley 1 and 2 correspond to the valleys at around 398 nm and 550 nm, respectively. Peak 1 and 2 correspond to the peaks at around 466 nm and 675 nm, respectively. The values of p-v 1 and 2 are equal to (peak 1)/(valley 1) and (peak 2)/(valley 2), respectively. It is obvious that at the beginning the p-v ratios are enlarged with the increase of Ag sputtering time, in accordance to the augment of Ag layer's thickness. However, when the thickness is too large, the p-v ratio will decrease instead. Here t_a is the thickness of Ag coating layer. The data in this table is obtained from Fig. 2b and Supplementary Fig. 8b.

Supplementary Table 2 Corresponding etching time of samples displayed in Fig. 3a.

138.5	135	131.5	128	124.5	121
117.5	114	110.5	107	103.5	100
96.5	93	89.5	86	82.5	79
75.5	72	68.5	65	61.5	58
54.5	51	47.5	44	40.5	37
33.5	30	22.5	15	7.5	0

The unit is minute. The relative positions of these etching times are in accordance to the positions of their corresponding samples demonstrated in Fig. 3a.

Supplementary Notes

Supplementary Note 1 The theory of pristine phase modulation in three layer system.

When a plane wave is normally incident to an interface of air and a dielectric thin film (thickness = L) on a substrate, its reflective and refractive behaviors can be approximately described as the situation in Supplementary Scheme 1. According to Fresnel formulae[1], the reflection and transmission coefficients can be expressed as

$$r_{ab} = \frac{n_a - n_b}{n_a + n_b} \quad (1)$$

$$t_{ab} = \frac{2n_a}{n_a + n_b} \quad (2)$$

where ab represents the incident direction from dielectric a to dielectric b, and n_a represents the refractive index of dielectric a. The complex amplitudes of light waves reflected by interface 1 (E_{r1}) and interface 2 (E_{r2}) can be written in the form of

$$E_{r1} = B_1 E_1 = A_1 E_1 e^{i\varphi_1} \quad (3)$$

$$E_{r2} = B_2 E_1 = A_2 E_1 e^{i\varphi_2} \quad (4)$$

where $B_1 = r_{12}$, $B_2 = r_{23} t_{12} t_{21} e^{i2k_2 L}$, and A_1 and A_2 are real number. So the phase difference between E_{r1} and E_{r2} is given by

$$\Delta\varphi_{21} = \varphi_2 - \varphi_1 \quad (5)$$

which can be modulated by adjusting the thickness (L) and refractive index (n_2) of the thin film.

Subsequently, we can also theoretically calculate reflection of the incident light.

Generally, light field in the three layer system with normal incident can be expressed as

$$E(z)=\begin{cases}E_1\left(e^{ik_1 z}+re^{-ik_1 z}\right) & (z<0)\\ E_1 s\left(e^{ik_2 z}+\gamma e^{-ik_2 z}\right) & (0\le z\le L)\\ E_1 te^{i(k_3 z)} & (z>L)\end{cases} \tag{6}$$

where $r=\frac{(n_1-n_2)(n_2+n_3)+(n_1+n_2)(n_2-n_3)e^{i2k_2L}}{(n_1+n_2)(n_2+n_3)+(n_1-n_2)(n_2-n_3)e^{i2k_2L}}$, which is deduced by considering the boundary conditions of electromagnetic waves and nonferromagnetic materials[2]. Then the reflection of the incident light is given by

$$R_1=|r|^2 \tag{7}$$

From the calculated results of phase difference and light reflection, it is found that the reflective spectrum of this three layer system (one film on substrate) is tailored by the phase difference spectrum, which can be modulated by change the thickness of the dielectric thin film.

Supplementary Note 2 The theory of conjugate twin phase modulation in four layer system.

In the next step, we consider a four layer system with a thin coating layer (thickness = *h*) on the dielectric film discussed above. With normal incidence in this case, the reflective and refractive behaviors of light wave can be approximately described as the situation in Supplementary Scheme 2. The complex amplitudes of light waves reflected by interface 0 (E_{r0}), 1 (E_{r1}) and 2 (E_{r2}) can be written in the form of

$$E_{r0}=B_0E_0=A_0E_0e^{i\varphi_0} \tag{8}$$

$$E_{r1} = B_1 E_0 = A_1 E_0 e^{i\varphi_1} \quad (9)$$

$$E_{r2} = B_2 E_0 = A_2 E_0 e^{i\varphi_2} \quad (10)$$

where $B_0 = r_{01}$, $B_1 = r_{12} t_{10} t_{01} e^{i2k_1 h}$, $B_2 = r_{23} t_{10} t_{01} t_{12} t_{21} e^{i2(k_1 h + k_2 L)}$, and A_0, A_1 and A_2 are real number. So the phase difference between E_{r0} and E_{r2} and between E_{r1} and E_{r2} are given by

$$\Delta\varphi_{20} = \varphi_2 - \varphi_0 \quad (11)$$

$$\Delta\varphi_{21} = \varphi_2 - \varphi_1 \quad (12)$$

which can be modulated by adjusting the thickness and refractive index of these two thin film. Particularly, when the coating layer is ultrathin (i.e., h is close to 0), if n_1 and n_2 are all real number and $n_1 > n_2$ and $n_1 > n_0$, the waves incident into the coating layer (E_{1i}) and reflected back to the coating layer (E_{1r1}) are in phase ($\Delta\varphi \to 0$), and they are all out of phase ($\Delta\varphi = \pi$) with the waves reflected directly by the interface 0 (E_{r0}) because of the π phase transition in E_{r0}. The conjugate twin interference will happen in this particular case, where the CI (E_{1r1} with E_{1r2} and E_{1i}) inside the coating layer occurs when the DI of the reflected waves (E_{r0} with E_{r1} and E_{r2}) at interface 0 (outer surface of the structure) takes place.

To figure out the effects of conjugate twin phase modulation, the behaviors of light waves, including reflection and electric field intensity, also need to be investigated. Similar to the three layer system, light field in the four layer system with normal incident can be expressed as

$$E(z)=\begin{cases} E_0\left(e^{ik_0 z}+r_0 e^{-ik_0 z}\right) & (z<0) \\ E_0 a\left(e^{ik_1 z}+r e^{-ik_1 z}\right) & (0<z<h) \\ E_0 s\left(e^{ik_2 z}+\gamma e^{-ik_2 z}\right) & (h<z<h+L) \\ E_0 t e^{i(k_3 z)} & (z>h+L) \end{cases} \qquad (13)$$

where

$$r=\frac{(n_1-n_2)(n_2+n_3)+(n_1+n_2)(n_2-n_3)e^{i2k_2L}}{(n_1+n_2)(n_2+n_3)+(n_1-n_2)(n_2-n_3)e^{i2k_2L}}e^{i(k_2+2k_1)h} \qquad (14)$$

$$r_0=\frac{(1+r)n_0-(1-r)n_1}{(1+r)n_0+(1-r)n_1} \qquad (15)$$

$$a=\frac{1+r_0}{1+r} \qquad (16)$$

The reflection of the incident light is given by

$$R_1=|r_0|^2 \qquad (17)$$

The intensity of electric field at the bottom surface of the coating layer is given by

$$|E_1|=\left|a\left(e^{ik_1 h}+re^{-ik_1 h}\right)\right|E_0 \qquad (18)$$

Larger $|E_1|$ represents constructive interference while smaller $|E_1|$ represents destructive interference.

Supplementary References

1 Born, M. & Wolf, E. *Principles of Optics*, 7th ed. (Cambridge U. Press, 1999).

2 Jackson, J. D. *Classical Electrodynamics*, 3rd ed. (Wiley, 1999)

3 Palik, E. D. & Ghosh, G. *Handbook of Optical Constants of Solids*. (Academic, 1998).

4 Hoyt, L. New Table of the Refractive Index of Pure Glycerol at 20°C. *Ind. Eng. Chem.* **26**, 329–332 (1934).
